# Book Reviews

**Published:** 1902-08

**Authors:** 


### *BOOK REVIEWS.*

---

Minor Surgery and Bandaging. Including the Treatment of Fractures and Dislocations, the Ligations of Arteries, Amputation, Excisions and Resections, Intestinal Anastomosis, Operations upon Nerves and Tendons, Tracheotomy, Intubation of the Larynx, etc. By Henry R. Wharton, M.D. Fifth Edition, Enlarged and Thoroughly Revised, with 509 Illustrations. 12mo, pp. 621. Philadelphia and New York: Lea Brothers & Co. 1902. Price $3.00 net.

This book is so well known and popular as not to require extended notice. The new edition embraces all of the features of the former editions besides being considerably enlarged. It does not confine itself to minor surgery, but is really quite a complete manual of surgery in all of its branches. It is very fully illustrated and is certainly to be recommended to every general practitioner.

---

The Freethinkers' Manual. By Prof. Dr. Baur, Physiologist, and H. F. Herbert, Electrician, and a great number of Scientists. Radical Publishing Co., Philadelphia, Pa., 1902.

This book covers widely the field of speculative thought, but views it with a jaundiced eye. If free thinking means freedom from religious trammels it might also mean freedom from sense. At any rate, it is not very wise or very safe to treat with levity things that are sacred to many and dear to many others. There are certainly many novel things in this book, but on the whole there can be no profit in reading it.

---

Diagnosis of Surgical Diseases. By Dr. E. Albert, Vienna. Translated from German (8th edition) by Robert T. Frank, A.M., M.D. D. Appleton & Co., N. Y., 1902.

Works on surgical diagnosis are comparatively few and perhaps for that reason a translation of one is justified. The work seems to us to be too narrow in scope. Many things are omitted and others superficially treated. The burning question of appendicitis is disposed of in two or three pages and that under the head of abdominal abscess. There is nothing about the prostate. The illustrations are very scanty. Otherwise the book is a fine volume.

